# Spatiotemporal characteristics of cortical activities of REM sleep behavior disorder revealed by explainable machine learning using 3D convolutional neural network

**DOI:** 10.1038/s41598-023-35209-1

**Published:** 2023-05-22

**Authors:** Hyun Kim, Pukyeong Seo, Jung-Ick Byun, Ki-Young Jung, Kyung Hwan Kim

**Affiliations:** 1 Department of Biomedical Engineering, College of Health Science, Yonsei University, Wonju, South Korea; 2 Department of Neurology, Kyung Hee University Hospital at Gangdong, Seoul, South Korea; 3 Department of Neurology, Seoul National University Hospital, Seoul National University College of Medicine, Seoul, South Korea

**Keywords:** Predictive markers, Sleep disorders

## Abstract

Isolated rapid eye movement sleep behavior disorder (iRBD) is a sleep disorder characterized by dream enactment behavior without any neurological disease and is frequently accompanied by cognitive dysfunction. The purpose of this study was to reveal the spatiotemporal characteristics of abnormal cortical activities underlying cognitive dysfunction in patients with iRBD based on an explainable machine learning approach. A convolutional neural network (CNN) was trained to discriminate the cortical activities of patients with iRBD and normal controls based on three-dimensional input data representing spatiotemporal cortical activities during an attention task. The input nodes critical for classification were determined to reveal the spatiotemporal characteristics of the cortical activities that were most relevant to cognitive impairment in iRBD. The trained classifiers showed high classification accuracy, while the identified critical input nodes were in line with preliminary knowledge of cortical dysfunction associated with iRBD in terms of both spatial location and temporal epoch for relevant cortical information processing for visuospatial attention tasks.

## Introduction

Rapid eye movement sleep behavior disorder (RBD) is a parasomnia characterized by sleep interruption and dream enactment. Isolated/idiopathic RBD (iRBD) occurs in the absence of neurological symptoms and represents a prodromal stage of neurodegenerative disorder^1^. More than 70% of iRBD patients develop severe neurodegenerative disorders, such as Parkinson’s disease and dementia with Lewy bodies, within 10 years^2,3^. Cognitive dysfunction, including executive function, episodic memory, and visuospatial perception, are observed in patients with iRBD^4,5^. Gagnon et al. reported that half of their patients had mild cognitive impairment^6^.

Determining the neural basis of cognitive impairment of iRBD patients with iRBD may provide important information for early intervention strategies for neurodegenerative disorders. The purpose of this study was to reveal the spatiotemporal characteristics of the cortical activity of patients with iRBD, which distinguish them from normal controls, and to discover neuromarkers reflecting abnormal cortical activities based on single-trial event-related electroencephalography (EEG) during an attention task.

Recent advances in machine learning, especially deep neural networks, have also been applied to high-density EEG analysis^7^ and have resulted in significant progress in several applications such as motor imagery, seizure detection, and sleep stage classification^8–11^. Many of these studies are based on convolutional neural networks (CNN), which mimic the characteristics of the central visual system and effectively utilize the structural information of the input data to reveal the underlying information^12^. CNN is particularly successful in image processing and computer vision^13^; thus, two-dimensional CNN (2dCNN) is mostly adopted^14,15^. However, visual data essentially represent both spatial and temporal information and are three-dimensional (3d). 3dCNN has recently been applied to hand motion video for hand gesture recognition^16,17^, and airport video for human action recognition^18^. Our data, multichannel EEGs, can be converted to current density time series on cortical surfaces using source localization techniques^19^, which are essentially 3d spatiotemporal data.

In a recent study, we identified the spatial characteristics of dysfunctional cortical activities of patients with neurological disorders^20,21^ based on a 2dCNN trained by 2d data representing current densities on the cortical surface within a critical temporal period, which is supposed to be crucial for working memory^22^. The temporal period was determined based on prior knowledge of the cognitive function under consideration, which may be misleading and has resulted in limitations in the objective identification of crucial characteristics solely based on a data-driven approach.

Here, we tried to discriminate cortical activities of iRBD patients from normal controls during cognitive function using 3dCNN, and to localize critical spatial location and temporal epoch, which reflects dysfunctional cortical activities associated with iRBD, by applying an explainable machine learning approach, that is, by identifying the input nodes of the CNN that play critical roles in the decision of the output. It is expected that the proposed method will contribute to elucidating the neural mechanism of abnormal brain activity in patients with iRBD, which cannot be revealed by conventional statistical analysis. Compared with our previous approach using 2dCNN, the 3dCNN-based method proposed here relies entirely on the data, without an a priori assumption on the critical temporal epoch.

## Methods

### Subjects and clinical screenings

A detailed description of the experimental procedures is presented in our previous paper^23^, and is briefly summarized here. Drug-naïve iRBD patients who visited Seoul National University Hospital were enrolled in this study. Normal controls without any sleep-related symptoms or neuropsychological diseases were screened via a survey and clinical interview. Experimental data were collected from 49 iRBD patients (aged 65.96 ± 5.94, 29 males) and 49 normal controls (aged 66 ± 6.37, 33 males). All experimental procedures performed in this study were approved by the Seoul National University Hospital Institutional Review Board (IRB Number 1406-100-589). All experiments were performed in accordance with relevant guidelines and regulations. Informed consent was obtained from all the subjects.

The subjects underwent neurological and cognitive tests before the main experiment. RBD symptom severity was evaluated using the Korean version of the RBD screening questionnaire (RBDQ-HK)^24^. Autonomic dysfunction was assessed using the Scales for Outcomes in Parkinson’s Disease for autonomic symptoms (SCOPA-AUT)^25^. Sleep quality was assessed by using the Pittsburgh Sleep Quality Index (PSQI)^26^. Excessive daytime sleepiness was assessed using the Epworth Sleepiness Scale (ESS)^27^. Global cognitive function was evaluated using the Korean version of the Montreal Cognitive Assessment (MoCA)^28^ and Mini-Mental State Examination (MMSE)^29^.

The subject demographics and cognitive test results are presented in Table 1. No significant difference between iRBD patients and normal controls was found in demographics, except for education. Patients showed significantly higher SCOPA-AUT and PSQI scores. The neuropsychological test results (Table 2) revealed that MMSE, MoCA total, attention, abstraction, memory recall, and orientation scores were significantly lower in iRBD patients than in normal controls (Table 2).

**Table 1:** Demographics and questionnaires results.

	Control (n = 49)	iRBD (n = 49)	p-value	Cohen’s d
Age (years)	66.00 ± 6.37	65.96 ± 5.94	0.974	− 0.01
Sex^ϯ^	M: 33, F: 16	M: 29, F: 20	0.529	
Education	13.76 ± 2.70	12.14 ± 3.94	**0.036**	− 0.43
RBDQ-KR	4.94 ± 3.36	47.53 ± 19.46	**< 0.001**	3.05
SCOPA-AUT	5.88 ± 4.40	13.02 ± 7.76	**< 0.001**	1.13
PSQI	3.39 ± 1.55	6.92 ± 4.31	**< 0.001**	1.09
ESS	4.2 ± 2.74	5.27 ± 3.32	0.087	0.35

Data are presented as mean ± standard deviation.

*RBDQ-KR* Korean version of the RBD screening questionnaire-Hong Kong, *SCOPA-AUT* scales for outcomes in Parkinson’s disease-autonomic, *PSQI* Pittsburgh sleep quality index total, *ESS* epworth sleepiness scale.

^ϯ^Fisher’s exact test.

p-value: independent t-test.

**Table 2:** Neuropsychological assessment.

	Control (n = 49)	iRBD (n = 49)	p-value	Cohen’s d
MoCA total	27.45 ± 1.49	25.57 ± 3.04	**< 0.001**	− 0.78
Visuospatial/executive	4.57 ± 0.68	4.41 ± 0.84	0.292	− 0.21
Naming	2.9 ± 0.31	2.78 ± 0.42	0.103	− 0.33
Attention	5.78 ± 0.47	5.31 ± 0.82	**< 0.001**	− 0.70
Sentence repetition	2.8 ± 0.46	2.59 ± 0.64	0.073	− 0.37
Abstraction	1.98 ± 0.14	1.82 ± 0.39	**0.007**	− 0.55
Memory recall	3.37 ± 0.95	2.69 ± 1.5	**0.009**	− 0.54
Orientation	5.98 ± 0.14	5.78 ± 0.62	**0.027**	− 0.45
MMSE	28.88 ± 1.29	27.27 ± 2.14	**< 0.001**	− 0.91

Data are presented as mean ± standard deviation.

*MoCA* montreal cognitive assessment, *MMSE* mini-mental state examination.

p-value: independent t-test.

Significant values are in bold.

Subjects performed Posner’s cueing task while multichannel EEG signals were being recorded^30^. In every single-trial, a cue stimulus was presented on the left or right side of the central fixation point, and then a target stimulus was presented in the same (valid) or opposite (invalid) position. The time interval between the cue and the target stimulus was either 200 ms (SOA 200 condition) or 1000 ms (SOA 1000 condition). Subjects were asked to press a button as soon as possible in response to the target stimuli. Five hundred trials were presented to subjects.

### EEG acquisition and preprocessing

Sixty-channel EEGs with a sampling frequency of 400 Hz were recorded based on 10–10 system. Two electrooculogram channels were placed on the left and right outer canthi to remove eye-related artifacts. Reference and ground electrodes were placed on the ear and AFz sites, respectively. The electrode impedances were maintained at below 10 kΩ. The acquired EEG signals were band-pass filtered for a frequency range of 0.1–70 Hz along with a 60 Hz notch filter. The recorded signals were re-referenced to the average of all the electrodes. Single-trial EEG, which is heavily contaminated by signal drift, high amplitude above 100 μV, and non-stationary noise with high-frequency fluctuations, was removed by visual inspection. Stationary noise, such as eye and muscle artifacts, was corrected using independent component analysis^31^.

### Data analysis

**Figure 1:**
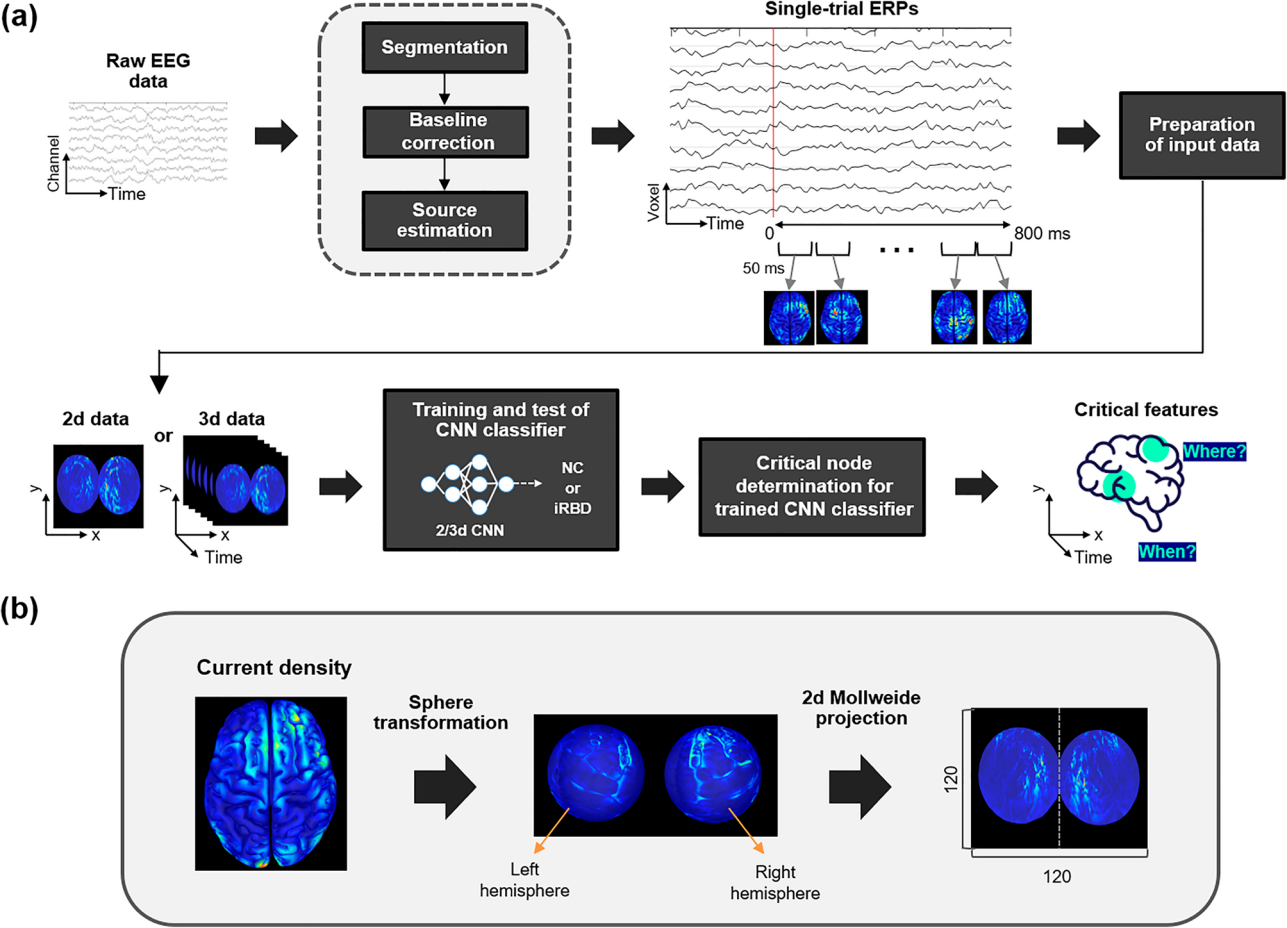
Overall procedure for the data analysis. (**a**) Data analysis pipeline including single-trial waveform segmentation, current source density estimation, CNN classifier, and determination of critical spatiotemporal characterization. (**b**) Detailed illustration of the projection of the current densities on a cortical surface onto a flattened 2d surface.

The overall procedure for the data analysis is presented in Fig. 1. The preprocessed EEG signals were segmented into single-trial waveforms based on the target stimulus onset (− 1200 to 800 ms). Multichannel EEGs were transformed to cortical current density time series by weighted minimum norm estimation (wMNE)^32^ cortical source estimation, which yielded 3d input data for the 3dCNN classifier. After successful training, critical input nodes were identified so that the spatial and temporal characteristics of cortical activity significantly reflected the difference between patients with iRBD and normal controls. A similar procedure was performed using the 2dCNN for comparison. In this case, the critical temporal period was predetermined to be 200–350 ms, which is known to be important for visuospatial attention^33^. The cortical current density time series were converted into 2d images by averaging within this critical period.

### Preparation of input data for CNN classifier

3d input data were constructed by concatenating 2d images of the cortical current densities over multiple temporal points, as shown in Fig. 1A. After segmentation during − 1200 to 800 ms intervals, further lowpass filtering (< 30 Hz) and baseline correction were performed by subtracting the average amplitude between − 200 and 0 ms. EEG recordings were converted to current density time series over 0–800 ms on 15,002 equally-distributed points on cortical surfaces using the Brainstorm toolbox^34^. For the forward problem, a volume conduction model was constructed from the ICBM 152 anatomical template, which is a distributed boundary element method^35^. Weighted minimum norm estimation was applied to estimate the current source density distribution, as explained by Tadal et al.^36^.

The 15,002 points on the cortical surface were first projected onto a sphere with registered coordinates in Brainstorm, and then the surface of the sphere was further projected onto a 2D plane using the Mollweide projection (Fig. 1B)^37,38^. For each time point, a 2D image of the cortical current sources was generated by interpolating the values at 15,002 points onto an equally spaced 120 × 120 uniform grid. The pixel intensities of the 2D images were converted to z-scores via standardization. Then, the current densities within 50 ms epochs were averaged, resulting in 16 2d images during 0–800 ms. Thus, the dimension of the 3d input to the CNN was 120×120×16. Totally 47,513 3d data were generated. Of these, 23,553 were from 49 normal controls, and 23,960 were from 49 patients with iRBD. For the 2dCNN, the dimension of the data was 120 × 120 × 1, since a 2d image of cortical current density was obtained by averaging within 200–350 ms. This temporal epoch is known to be critical for visuospatial attentional processing during the Posner task, corresponding to N1 and P300 event-related potential (ERP) components^23,33,39^.

### The structure of CNN classifier

**Figure 2:**
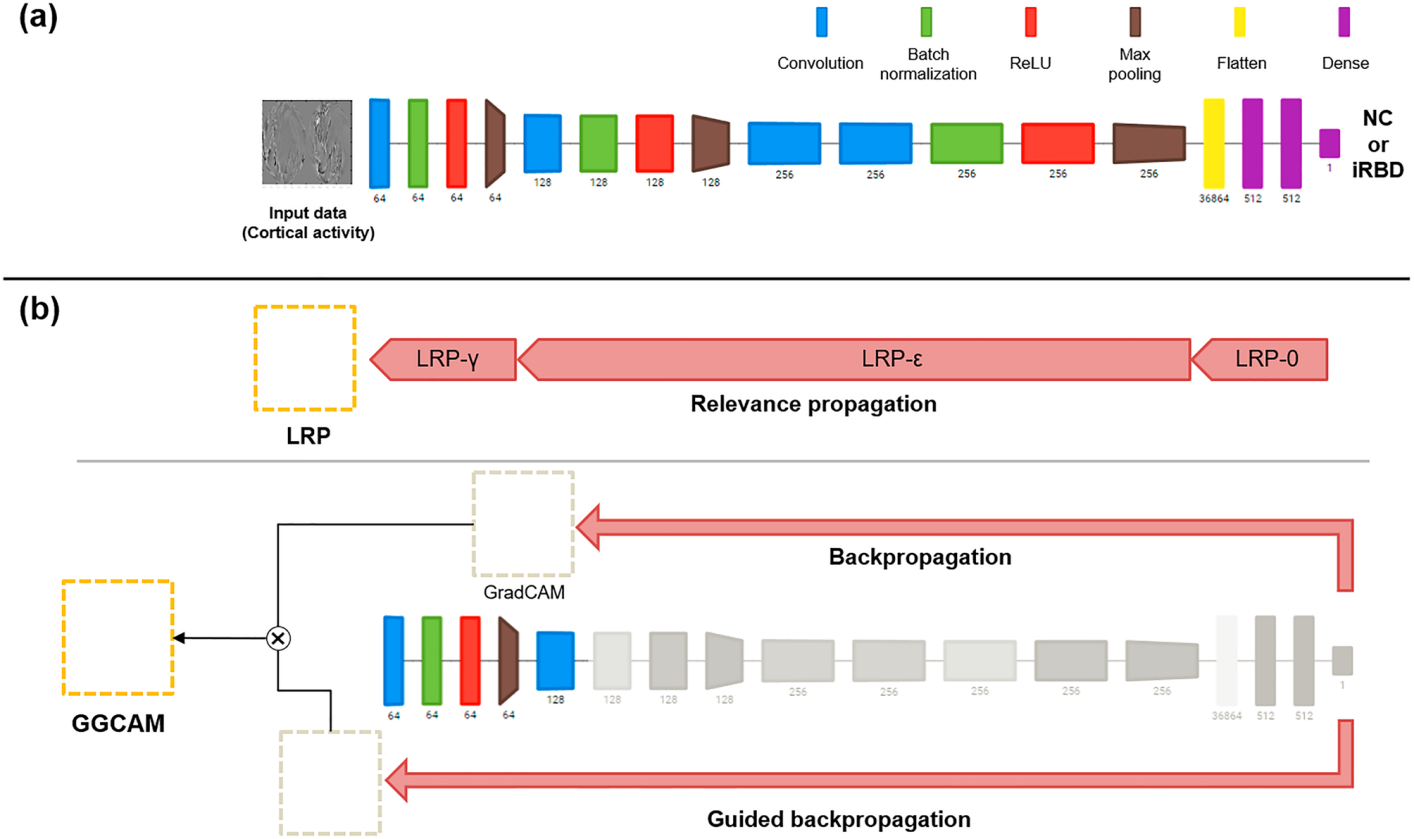
(**a**) The structure of the CNN classifier. The type of layer is depicted by color of each block (blue: a convolutional layer, green: a batch normalization layer, red: an activation layer, brown: a max pooling layer, yellow: a flatten layer, purple: a fully connected layer). The filter size is denoted below each block. (**b**) Methods for the determining the critical input nodes, based on LRP (upper) or GGCAM (lower).

The structure of the CNN classifier was devised based on the C3D model, which has been shown to be effective in learning spatiotemporal features from 3d video data^40,41^. The convolution module in the CNN consists of three repetitions of a convolutional layer, batch normalization layer, and max pooling layer, followed by two fully connected layers and one output layer that performs classification, as shown in Fig. 2A. The filter sizes of each convolution module were 64 µm, 128 µm, and 256 µm. The structures of the 2dCNN and 3dCNN classifiers are identical, except for the type of convolutional layer, filter size, and stride size.

The kernel sizes of the convolutional layers of 3dCNN were 3 × 3 × 3, with stride sizes of 1. The max pooling layers had kernel and stride sizes of 2 × 2 × 2, except for the first layer. The kernel/stride size of the first max-pooling layer was 2 × 2 × 1.

For the 2dCNN, all convolutional layers had a kernel size of 3 × 3, with a stride size of 1. The pooling layers were max-pooling layers with kernel and stride sizes of 2 × 2. The filter size of the fully connected layer is 512. The activation functions for all nodes were rectified linear functions, except for the output layer nodes, for which the sigmoid activation function was adopted.

In addition, we performed an analysis of performance changes according to the depth of the network, including learning accuracy and robustness. Three structures were tested: shallow, standard, and deep (Fig. S1).

### Training and test of the classifier

**Figure 3:**
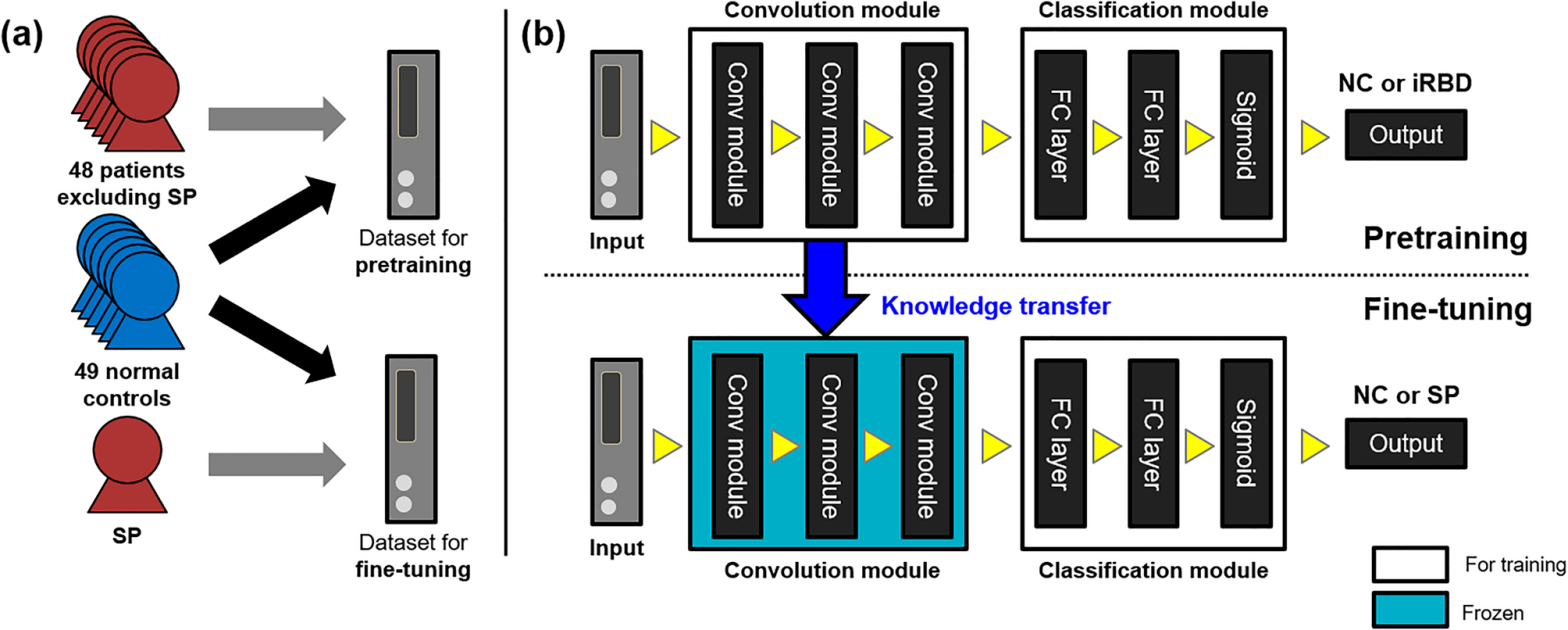
Transfer learning for the training of the classifiers. (**a**) Construction of the input data for the CNN classifiers for the pretraining and fine-tuning. (**b**) Pretraining based on the data excluding a specific patient (SP) and Fine-tuning of the classifier for the SP, based on the data from the SP and normal controls.

The training and evaluation of the CNN classifier consisted of two stages: pretraining and fine-tuning/evaluation, as shown in Fig. 3. First, the training data were prepared by eliminating all data from a single specific patient (SP) for pretraining. After successful pretraining, a transfer learning approach was applied to the SP, and the classification accuracy was evaluated. This procedure was repeated for all the 49 patients with iRBD. Training and testing of the CNN were performed using an AMD Ryzen Threadripper 2990WX 32-Core Processor, four Nvidia GeForce RTX 2080 Ti graphics cards, 128 GB access memory, and an open-source machine learning library, PyTorch Lightning package^42^. For the 2dCNN classifier with 20.1M parameters, the number of floating-point operations (FLOPs) were 104.07B for a batch size of 128. The training time per subject was 3950.59 ± 3508.92 s for the pretraining stage, and 649.47 ± 140.82 s for the fine-tuning stage. For the 3dCNN classifier with 22.02M parameters, the FLOPs were 1727.88B for a batch size of 128. The training time per subject was 7177.02 ± 1783.30 s for the pretraining stage, and 2194.61 ± 1234.42 s for the fine-tuning stage.

#### Pretraining stage

A CNN classifier was trained on the dataset from 97 subjects, excluding the SP (the upper part of Fig. 3B). Random undersampling was applied to avoid the class imbalance problem so that the ratio of data points in the two classes (iRBD patients and normal controls) was 1:1^43^. The data were further divided randomly into training (90%) and validation (10%) sets. The weights and biases were initialized using the Kaiming method^44^. Owing to limited memory allocation capacity, the mini-batch size was set to 128. The binary cross-entropy loss function was adopted and minimized using the Adam optimizer^45^. The optimal learning rate was determined to be within the range of 1 × 10^−8^ to 1 using the learning rate range test proposed by Smith^46^. The weight decay was set as 1×10^−5^. The classifier was trained for 100 epochs and early stopping was applied if the validation accuracy did not improve after 10 epochs.

#### Fine-tuning and evaluation stage

For the fine-tuning of each SP, the input data for the training were constructed from the data of the SP and randomly selected data from normal controls that were not used for the pretraining, as shown in Fig. 3A. Data from healthy controls were included to avoid overfitting to a single class (iRBD patient class). 80% of the dataset was used for training, and 20% was used for the evaluation. During the training for the fine-tuning, only the parameters of the fully connected layers and output layer were adjusted, whereas those of the convolution layers were fixed to those obtained from the pretraining stage (the lower part of Fig. 3B). The convolution layers of the pretrained model are known for their ability to extract useful features from images that can be used for various image classification tasks^47,48^. Therefore, the convolutional layer is frozen to retain the pre-learned features, and only the fully connected layer is allowed to learn task-specific features from unseen patient data. However, if the new data differ significantly from the data used in the pretrained model, or if the fully connected layer does not learn task-specific features effectively, the convolutional layer can be fine-tuned to fit the new data. The learning rate and weight decay were set to 1/10 of the pretraining values to prevent overfitting^49,50^. All other parameters were set to be equal to those for pretraining.

### Determination of critical input features

Spatiotemporal characteristics of neural activity reflecting distinct difference between iRBD patients and normal controls were identified by finding the nodes in the input layer which contribute considerably to the decision of the CNN classifiers, i.e, by ‘explaining’ the CNN. Two representative methods for the ‘explainable machine learning,’ layer-wise relevance propagation (LRP) and guided gradient-weighted class activation mapping (GGCAM), were adopted here^51,52^ (Fig. 2B).

LRP is a method for computing the relevance scores of the nodes in the input layer by repeated backpropagation, which decomposes a single node’s output into the contributions of the nodes in the previous layer. Backpropagation for the relevance scores is performed as shown below in Eq. (1).

1 $${R}_{j}^{l}=\sum_{k}\frac{{z}_{jk}}{{\sum }_{j}{z}_{jk}}{R}_{k}^{l+1}$$

where $l$ denotes the number of layers. ${R}_{k}^{l+1}$ indicates the relevance of $k$ node in a higher layer, ${R}_{j}^{l}$ indicates the relevance of $j$ node in the lower layer. ${z}_{jk}$ denotes the influence of $k$ neuron of the higher layer on $j$ neuron of the lower layer.

Several improvements in the propagation rule of Eq. (1) have been presented^53^. We applied the LRP0 and LRP-gamma rules for the fully connected and convolutional layers, respectively, as proposed by Montavon et al.^51^. The source code for LRP is available at http://heatmapping.org. The set of relevance scores for the input nodes provided a heatmap representing the contribution of each cortical point to the classifier output.

Gradient-weighted class activation mapping (Grad-CAM) is a method used to find the nodes that contribute greatly to the output based on the gradient of the output with respect to their activation^52^. For 3dCNN, the importance score of a node $ijk$, ${L}_{ijk}$, is calculated as the product of its activation ${A}_{ijk}^{n}$ and the average of the class score gradient of the feature map to which node $ijk$ belongs (denoted by $n$), as follows:

2 $${L}_{ijk}=ReLU\left(\sum_{n}{w}_{n}\times {A}_{ijk}^{n}\right)$$

where,

3 $${w}_{n}=\frac{1}{Z}\sum_{i}\sum_{j}\sum_{k}\frac{\partial y}{\partial {A}_{ijk}^{n}}$$

where $y$ denotes the output from the output layer, which corresponds to the class score. where $i$, $j$, and $k$ represent the indices for the location of a node in a 3d feature map. For the 2dCNN classifier, the score of node $ij$ is calculated in the same manner.

Guided GradCAM (GGCAM) was proposed by Selvaraju et al.^52^ to alleviate the problem of low resolution of GradCAM, which obtains the heatmap at the middle layer. A method called guided backpropagation (GBP) is applied here to achieve the resolution of the input layer after upsampling the heatmap obtained by GradCAM (Eq. (3)) to the size of the input layer. GBP refers to an algorithm that calculates the gradient of the class score with respect to the network parameters in the same way as a typical BP algorithm, except for backpropagation at the ReLU nodes^54^. BP and GBP can be described by Eqs. (4) and (5), respectively, as follows:

4 $${g}_{i}^{l}=\left({A}_{i}^{l}>0\right)\cdot {g}_{i}^{l+1}$$

5 $${g}_{i}^{l}=\left({A}_{i}^{l}>0\right)\cdot \left({g}_{i}^{l+1}>0\right)\cdot {g}_{i}^{l+1}$$

Here, $l$ and ${g}_{i}^{l+1}$ denote the layer number and the gradient of a node $i$ in a higher layer $l+1$. ${A}_{i}^{l}$ is the activation of node $i$ in lower layer $l$.

As shown in Eqs. (4) and (5), the gradient is not propagated to the lower layer if either the activation of the lower layer or the gradient of the higher layer is negative for the GBP, whereas it is not backpropagated only when the activation is negative for a normal BP. The GBP is repeated up to the input layer, and then the GGCAM heatmap is obtained at the resolution of the input layer by multiplying the GradCAM heatmap and the gradient obtained by Eq. (5).

### Statistical analysis

In this study, we calculated Pearson's correlation coefficients to examine the association between the cortical current density averaged over critical spatiotemporal regions and clinical/cognitive function scores^55^. A one-tailed test was performed to evaluate the strength of this relationship. Based on the subject demographics and cognitive test results, we hypothesized that critical spatiotemporal features would exhibit a negative correlation with clinical scores and a positive correlation with cognitive function scores.

## Results

### Classification performance

**Figure 4:**
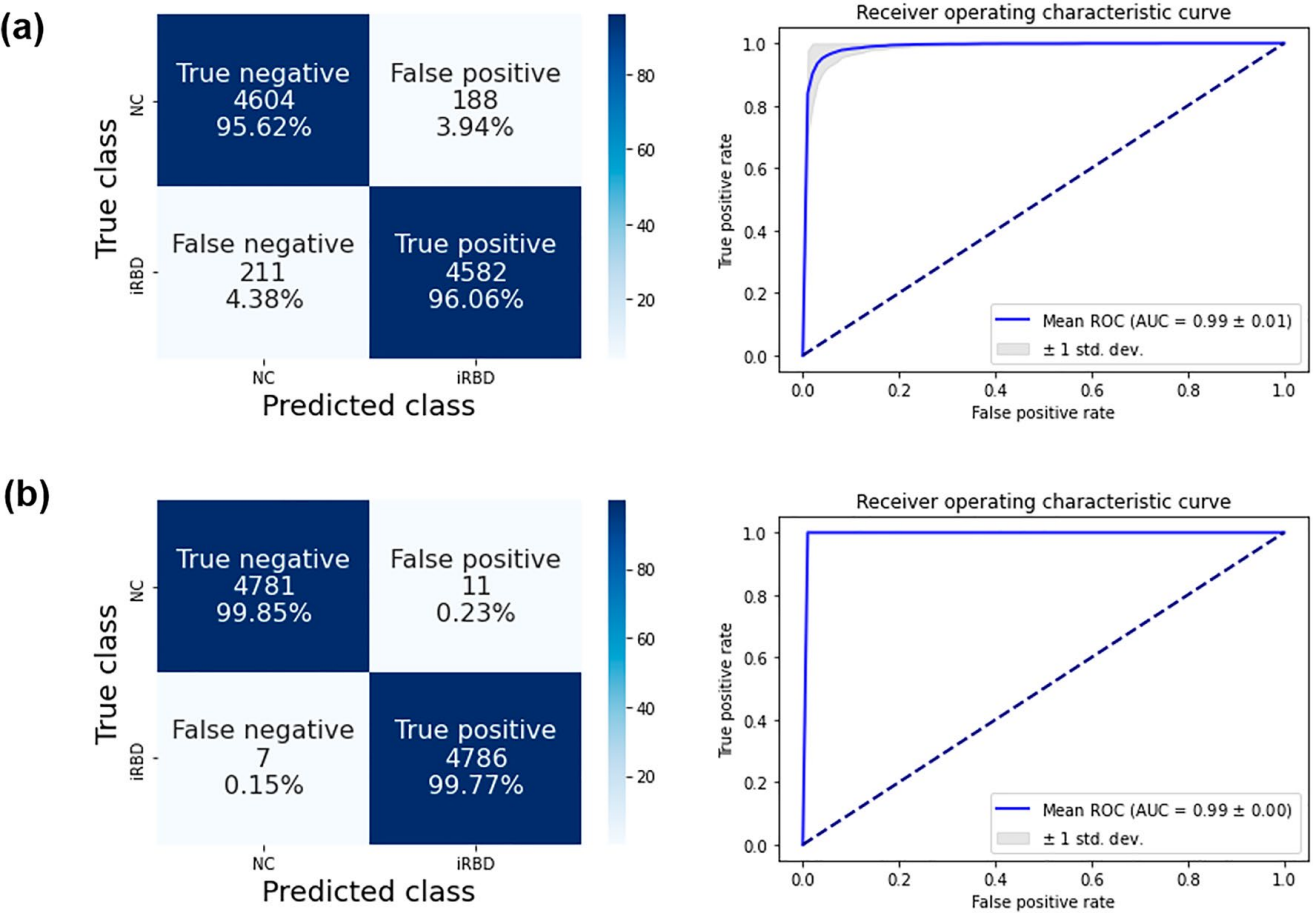
Classification accuracies for the evaluation data from all the SPs. (**a**) Confusion matrix and ROC for the 2dCNN classifier. (**b**) Confusion matrix and ROC for the 3dCNN classifier.

For the 2dCNN classifier, the training accuracy was 99.26 ± 0.62% for pretraining and 100.00 ± 0.02% for fine-tuning. The evaluation on test data showed the mean test accuracy of 95.83 ± 2.41% (precision 96.12 ± 2.72%, recall 95.59 ± 3.73%, AUROC 98.80 ± 0.63%). Figure 4 presents the classification accuracies for the evaluation data from all SPs. The left panel of Fig. 4A shows a confusion matrix that summarizes the classification results of the 2dCNN classifier. The true positive rate for iRBD patients was 96.06% and the true negative rate for the normal controls was 95.62%.

For the 3dCNN classifier, the training accuracy was 100 ± 0.00% for both the pretraining and fine-tuning stages. The evaluation on the test data showed the mean accuracy of 99.81 ± 0.32% (precision 99.77 ± 0.47%, recall 99.85 ± 0.47%, and AUROC 99.49 ± 0.01%). The left panel of Fig. 4B shows a confusion matrix summarizing the classification results for the 3dCNN classifier. The true positive and true negative rates were 99.77% and 99.85%, respectively, which demonstrated lower errors compared with the 2dCNN classifier. A statistical comparison of the 2d and 3dCNN classifiers showed that the classification performance of 3dCNN was significantly higher than that of 2dCNN (t(48) = 11.50, p < 0.001).

The classification performance was not significantly different among the structures, except that the training accuracy of the shallow structure increased slowly with respect to the number of iterations (Fig. S2).

### Critical spatiotemporal features of cortical activity

**Figure 5:**
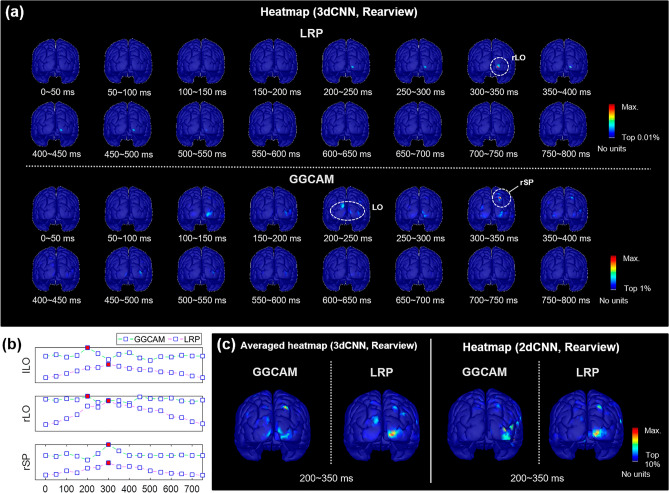
The distribution of relevance scores on cortical surface for the correctly classified data. (**a**) Heatmaps of the relevance score obtained from the 3dCNN classifiers at multiple temporal windows (50 ms-wide). (**b**) The change of relevance scores over time for the three ROIs denoted by white dotted ellipse in (**a**). The maximum scores are denoted by red squares. (**c**) Heatmaps of the relevance scores obtained from 3dCNN (left) and 2dCNN (right) at a critical temporal epoch (200–350 ms). This figure was created using Brainstorm (http://neuroimage.usc.edu/brainstorm) and MATLAB R2020a (MathWorks, Natick, Massachusetts, USA).

The heatmaps in Fig. 5 present the distribution of relevance scores on the cortical surface (rearview) at 50 ms time intervals obtained by averaging the correctly classified test data from iRBD patients (rearview). The spatiotemporal distributions obtained by the two methods, LRP and GGCAM, were similar, that is, high scores were focused on similar spatiotemporal regions. Overall, the heatmaps obtained by 2dCNN were also close to those obtained by 3dCNN when the temporal epoch was carefully predetermined to 200–350 ms (Fig. 5C).

The critical cortical region revealed by 3dCNN + LRP was located around the right lateral occipital region (LO) at 200–500 ms, while 3dCNN + GGCAM yielded the bilateral occipital region at 100–400 ms, and the right superior parietal lobule (SPL) at 300–400 ms. The right LO was consistently identified in both LRP and GGCAM (Fig. 5A).

Figure 5B shows the change in relevance scores with respect to time for the three critical cortical areas. For the LO region, the GGCAM score was the highest at 200–250 ms, while the LRP score was the highest at 300–350 ms. Both methods showed the highest values at 300–350 ms for the right SPL region (Fig. 5B).

**Figure 6:**
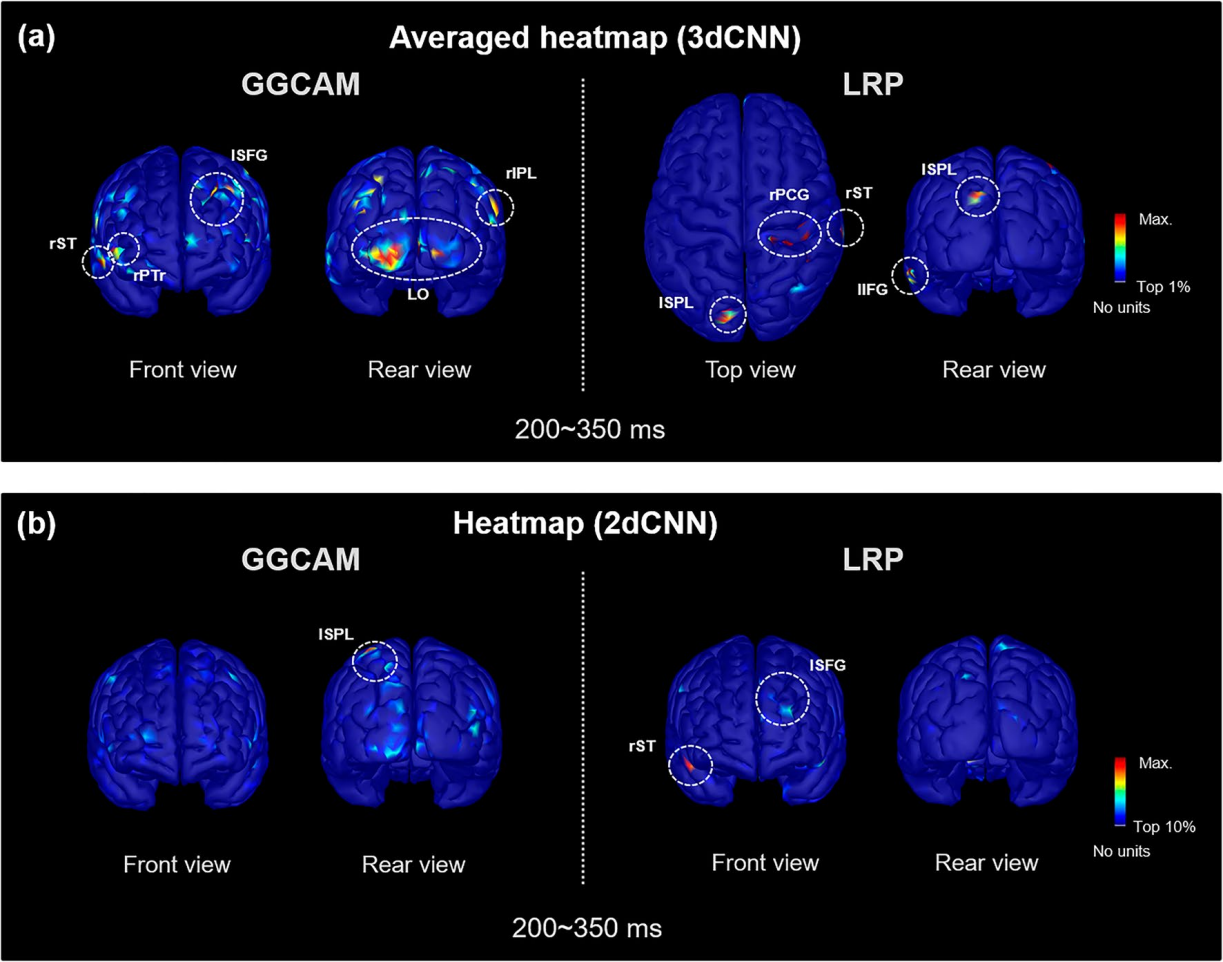
The distribution of relevance scores on cortical surface for the incorrectly classified data at a critical temporal period (200–350 ms). (**a**) Averaged heatmaps of the relevance scores obtained from 3dCNN. (**b**) Heatmaps of the relevance scores obtained from 2dCNN. This figure was created using Brainstorm (http://neuroimage.usc.edu/brainstorm) and MATLAB R2020a (MathWorks, Natick, Massachusetts, USA).

The heatmaps in Fig. 6 present the distribution of relevance scores for incorrectly classified data during the critical temporal period (200–350 ms). It is remarkable that the interpretation of the 2dCNN and 3dCNN classifiers is inconsistent, with different regions identified as important for the prediction, which is clearly different for the case of correctly classified data (Fig. 5). The heatmap analysis results were inconsistent across the LRP and GGCAM results.

We investigated the relationship between neural activity in the identified critical spatiotemporal ranges and cognitive function scores. Table 3 presents the results of the correlation analysis. Pearson’s correlation analysis showed that the average cortical current density in the right SPL region in the critical temporal range (Fig. 5B) was negatively correlated with the RBDQ-HK score (rho = − 0.17, p < 0.05). The average cortical current density in the right SPL region in the critical temporal range was significantly correlated with the MMSE score (rho = 0.26, p < 0.01) for all subjects. For iRBD patients only, the correlation was also significant (rho = 0.31, p < 0.05).

**Table 3:** Correlations between cortical current density averaged over the critical spatiotemporal regions and clinical/cognitive scores.

	200–250 ms left LO	300–350 ms left LO	200–250 ms right LO	300–350 ms right LO	300–350 ms right SPL
SCOPA-AUT	− 0.04	− 0.04	− 0.04	− 0.04	0.05
RBDQ-HK	0.02	0.02	− 0.07	− 0.07	− 0.168*
ESS	− 0.09	− 0.09	− 0.07	− 0.07	− 0.13
PSQI	− 0.10	− 0.11	0.05	0.04	− 0.06
MoCA total	− 0.10	− 0.09	0.02	0.02	0.14
Visuospatial/executive	− 0.184	− 0.183	0.00	0.00	0.02
Naming	0.05	0.05	0.06	0.06	0.16
Attention	− 0.03	− 0.03	0.00	0.00	− 0.03
Sentence repetition	0.01	0.01	0.00	0.01	0.02
Abstraction	− 0.10	− 0.10	0.02	0.02	0.14
Memory recall	− 0.01	0.00	0.04	0.04	0.04
Orientation	− 0.06	− 0.05	0.05	0.06	0.14
MMSE	0.03	0.03	0.14	0.14	0.264**

Data: Pearson’s correlation coefficient.

p-value: one-tailed test.

*p < 0.05, **p < 0.01.

## Discussion

In this study, we showed that the use of 3dCNN is advantageous for characterizing the differentiation of spatiotemporal neural activity between iRBD patients and normal controls, as it does not require any a priori assumptions on the critical location and time. These findings suggest that our 3dCNN-based approach may lead to the identification of useful neuromarkers for brain activity underlying the abnormal cognitive function associated with iRBD.

The interpretation method of the classifier produced a heatmap indicating the contribution of the cortical activity of each localized region in the spatiotemporal domain to the prediction of iRBD patients. We confirmed that the identified spatiotemporal information was correlated with cognitive function scores and consistent with neurophysiological profiles, thus determining it to be a neuromarker reflecting spatiotemporal attention impairment in patients with iRBD.

Conventional statistical techniques often involve comparing averaged single-trial EEGs between groups to identify ERP patterns. However, machine learning techniques can examine characteristic patterns in all single-trial EEGs without averaging them, uncovering subtle patterns that may not be visible through traditional statistical approaches, and preventing loss of information. In this study, we used an explainable machine-learning technique to identify spatiotemporal information that consistently contributes to the prediction of classifiers by averaging individual heatmaps. Future research can explore the variations among patients and trials by analyzing individual heat maps in greater depth.

Cortical activities in the bilateral LO at 200–350 ms and right SPL at 300–350 ms were found to be critical in discriminating iRBD patients from normal controls. The LO region receives visual inputs in a bottom-up manner and is modulated by top-down attention^56^, thus playing a pivotal role in visuospatial attentional processing triggered by target stimuli^57^. The 200–250 ms period coincides with the latency of the N1 ERP component, which is known to be devoted to early visuospatial processing^23^. Therefore, we estimate that the neural activity of the LO region during this period is devoted to early visuospatial processing for the attentional task and may underlie the differences in neurobehavioral responses between iRBD patients and normal controls.

During the 300–350 ms period, the LO and right SPL regions were found to be critical and are known to reflect higher-order visual processing, which is modulated by top-down control of spatial attention^58,59^. This is also consistent with previous results that showed right hemisphere dominance in visuospatial processing^60^. Our results may be interpreted as reflecting a higher cognitive load for visuospatial processing in iRBD patients than in normal controls.

We identified a significant negative correlation between cortical current density in the right superior parietal lobule (SPL) region and the RBDQ-HK score. Specifically, SPL activity was negatively correlated with the severity of RBD symptoms, suggesting that a decline in SPL activity may be related to an increase in symptom severity. For patients with iRBD, the MMSE score was highly correlated with SPL activity. Previous studies have shown that the SPL region is critical for spatial working memory and attention^61^, and especially for the spatial memory of cue location and attentional control for target stimuli processing during a visual search task. Thus, it is expected that the decreased SPL activity during the 300–350 period underlies the cognitive decline of iRBD patients.

Both methods for the interpretation of the trained classifiers, LRP and GGCAM, yielded similar results in terms of the spatial and temporal locations of critical regions. For the LO region, there was a slight difference in the temporal epochs (LRP: 300–350 ms, GGCAM: 200–250 ms). The LRP results are based on the relative contribution of each node in the input layer to the output, whereas GGCAM scores the positive gradient of the output with respect to the activity of each input node. Thus, we interpret that LO activity during 200–350 ms is critical for the differentiated cognitive function associated with iRBD. The output for the classification was most sensitive to the earlier activity (200–250 ms), which is expected to be devoted to early visual perception, whereas the later activity (300–350 ms), which is expected to underlie visuospatial attention, greatly contributed to determining the classifier output.

Both 2dCNN and 3dCNN provided similar results in that the heatmaps showed similar spatial distributions when the temporal epoch was predetermined based on previous knowledge of cortical activities for visuospatial attentional processing^23,33,39^. The spatial information provided by the method suggested in this study could be interpreted as representing cortical dysfunction for attentional processing associated with iRBD. The spatial characteristics of abnormal cortical activity associated with iRBD identified in the current study are consistent with the metabolic/hemodynamic profiles revealed by functional neuroimaging^42,43^. An FDG-PET study revealed abnormal metabolic network activities in patients with iRBD, characterized by decreased activities in occipital regions, including the lateral occipital region, lingual gyrus, and precuneus, and increased activity in the medial frontal region^62^. In addition, an fMRI study showed altered resting-state thalamocortical functional connectivity associated with cognition in iRBD^63^.

For correct prediction, spatiotemporal features reflecting cognitive impairment of the patients seem to play an important role in the judgement of the classifier, whereas the distribution of critical spatiotemporal features seems to be inconsistent and uninterpretable for incorrect prediction. This is in line with a previous study on diagnosing and interpreting patients with lung disease using chest X-rays^64^. When the patients were correctly classified, disease-related localized areas were identified as important features for judgement, whereas other irrelevant areas were identified as important features for incorrectly classified data. Furthermore, a study utilizing MRI to predict Alzheimer’s disease (AD) patients found that the features identified through interpretation methods in correctly predicted cases corresponded with the neuropathology of AD patients. Conversely, the features of incorrectly predicted cases are uninterpretable^65^.

In the case of 3dCNN, the critical temporal epoch was identified solely from the data without any a priori information and nearly coincided with the period assumed for the use of 2dCNN, which was based on previous ERP studies^23,33,39^. We also confirmed that the classification accuracy of the 2dCNN classifier was maximized when the temporal period was selected as that identified by the 3dCNN-based method. We expect that our results will provide a basis for further studies to identify the spatiotemporal characterization of the neural activity underlying abnormal cognitive function associated with various neurological/psychiatric disorders. Considering that the available screening methods for iRBD are rather limited in terms of both sensitivity and specificity (mostly below 85% accuracy)^66^, our methods based on the CNN classifier provide prospective alternative or supplementary tools for the screening of iRBD.

To verify whether the classifier was overly sensitive to small changes in the input data, we investigated the robustness of the classifier to noise by adding different noise levels to the input data. The experimental findings indicated that the proposed CNN classifier was unaffected by changes in the input data (Fig. S3). One way to assess a classifier’s generalization performance is to add noise to the input data. This technique learns more resilient features that are less sensitive to minor deviations in input data. However, it is worth noting that excessive noise can impede the classifier’s ability to recognize underlying patterns in the data, which may result in poor generalization outcomes. Hence, it is important to choose an appropriate noise level that is similar to the variations that the classifier may face in the clinical field.

For further analysis, we evaluated the generalization performance by cross-validating the model structure and adding different noise levels to the training data. The results confirm that our classifier is robust to noise and structure, resulting in low generalization error. In other words, we can conclude that the trained classifier has learned the underlying patterns and relationships in the data rather than simply memorizing the noise in the training data.

## Conclusion

Here, we presented methods to identify the spatiotemporal characteristics of abnormal cortical activities associated with iRBD underlying cognitive dysfunctions, especially during a visuospatial attention task, based on CNN classifiers and an explainable machine learning approach. By finding the important nodes in the input layer that contributed most significantly to the output after successful training of the classifiers, the critical spatiotemporal region could be determined, which is expected to represent the difference between patients with iRBD and normal controls. The 3dCNN based method is beneficial in that the data-driven approach can be implemented without any a priori assumptions with high accuracy.

Our method may contribute to further studies on the neural underpinnings of abnormal brain activity due to various neuropsychiatric diseases based on a relatively simple procedure using single-trial ERPs, which can be obtained from scalp EEG recordings.

## Supplementary Information

Supplementary Information.

## Data Availability

The data presented in this study are not publicly available because they contain information that can compromise the privacy of the research participants. Some of the data may be available from the corresponding authors upon request. The code and supplementary materials are available at GitHub: https://github.com/dosteps/iRBD_XML_3dCNN.
