# Supplementary material for: Spatiotemporal characteristics of cortical activities of REM sleep behavior disorder revealed by explainable machine learning using 3D convolutional neural network
Source: Sci Rep. 2023 May 22;13:8221. doi: 10.1038/s41598-023-35209-1 (PMC10202918; doi:10.1038/s41598-023-35209-1)
Supplement: Supplementary file 1 — Supplementary Information. [file 41598_2023_35209_MOESM1_ESM.docx]

**Spatiotemporal characteristics of cortical activities of REM sleep behavior disorder revealed by explainable machine learning using 3D convolutional neural network: supplemental document**

Hyun Kim1, Pukyeong Seo1, Jung-Ick Byun2, Ki-Young Jung3* & Kyung Hwan Kim1*

1*Department of Biomedical Engineering, College of Health Science, Yonsei University, Wonju, South Korea*

2*Department of Neurology, Kyung Hee University Hospital at Gangdong, Seoul, South Korea*

3*Department of Neurology, Seoul National University Hospital, Seoul National University College*

*of Medicine, Seoul, South Korea*

*** *correspondence: jungky@snu.ac.kr (K.-Y.J.); khkim0604@yonsei.ac.kr (K.H.K.)*

## Supplement 1: Classification accuracies for various CNN depths

The classification accuracies were examined while varying the depth of the CNN. 10-fold cross-validation was applied to the dataset used in the pretraining stage. Three structures were tested: shallow, standard, and deep (Fig. S1). The convolution module in the shallow CNN consisted of two repetitions of a convolutional layer, a batch normalization layer, and a max pooling layer, while they were repeated five times for the deep CNN.

The computational cost of classifiers exhibited significant variation depending on their type, rather than network depth. Notably, the 3dCNN classifier required about four times longer training time than the 2dCNN classifier. However, the variance in training time was considerable due to the implementation of early-stopping. Interestingly, the deep CNN showed slightly lower FLOPs than the standard CNN. This observation suggests that the deep CNN may have a more efficient structure that enables it to perform computations effectively despite having more layers. Moreover, the flattened layer had the most substantial impact on the size of network parameters. In the shallow CNN, the number of output nodes in the flattened layer was the largest (100,352), resulting in the highest network parameter size among the three structures.

The performance metrics of various CNN classifiers were evaluated to identify the impact of network depth and type on their computational efficiency. In the shallow CNN, the 2dCNN classifier with 51.72M parameters required 39.13B FLOPs for a batch size of 128, and the training time per cross-validation fold was 3602.4 ± 603.35 s. The 3dCNN classifier with 103.25M parameters had a significantly higher computational cost of 525.54B FLOPs and a longer training time of 10947.4 ± 4995.73 s.

For the standard CNN, the network parameters and the FLOPs were similar to those in the shallow CNN. However, the training time was shorter, with 2285.1 ± 698.63 s for the 2dCNN classifier and 11018.5 ± 4245.3 s for the 3dCNN classifier.

For the deep CNN, the 2dCNN classifier with 11.84M parameters required 102.02B FLOPs and had a training time of 2530.2 ± 730.31 s. The 3dCNN classifier with 13.76M parameters had a remarkably high computational cost of 1725.83B FLOPs, but the training time was relatively shorter at 8783 ± 2297.2 s. These findings suggest that network type, rather than depth, has a more significant impact on the computational cost of CNN classifiers.

The classification performance was not significantly different among structures, except that the training accuracy of the shallow structure increased slowly with respect to the iterations (Fig. S2). The training accuracies was quite high for the 2dCNN classifiers (shallow: 98.49 ± 0.58%, standard: 98.13 ± 0.63%, deep: 98.79 ± 0.23%). The validation accuracies were 85.4 ± 0.82% for the shallow, 84.62 ± 0.78% for the standard, 83.96 ± 0.59% for the deep CNN. The training accuracies for the 3dCNN classifiers were also very high (shallow: 99.78 ± 0.22%, standard: 99.80 ± 0.10%, deep: 99.81 ± 0.05%). The validation accuracies were significantly improved for the 3dCNN as compared to the 2dCNN, i.e., 98.49 ± 1.98%, 98.96 ± 0.56%, and 99.40 ± 0.22% for the shallow, standard, and deep 3dCNN classifiers, respectively.

## Supplement 2: Robustness of the proposed classifier to noise in the training data

We investigated the robustness of the classifier to noise by adding different levels of noise to the input data. The training and validation datasets for the pretraining stage were used to evaluate the training and validation accuracy of the 2dCNN classifier as a function of the noise level of the input data. First, the input data was standardized to z-score. Then, for every batch of training, zero-mean Gaussian noise was added to the input data, with 50% probability. The level of noise was varied by changing the variance of the noise (0, 0.01, 0.02, 0.05, 0.1, 0.2, 0.4, 0.6, 0.8, 1.0). Fig. S3A shows 2d input data after adding the noise. As shown in Fig. S3B, the classification accuracies were not substantially affected by the noise, although the speed of training was reduced for higher noise level.

# Figures


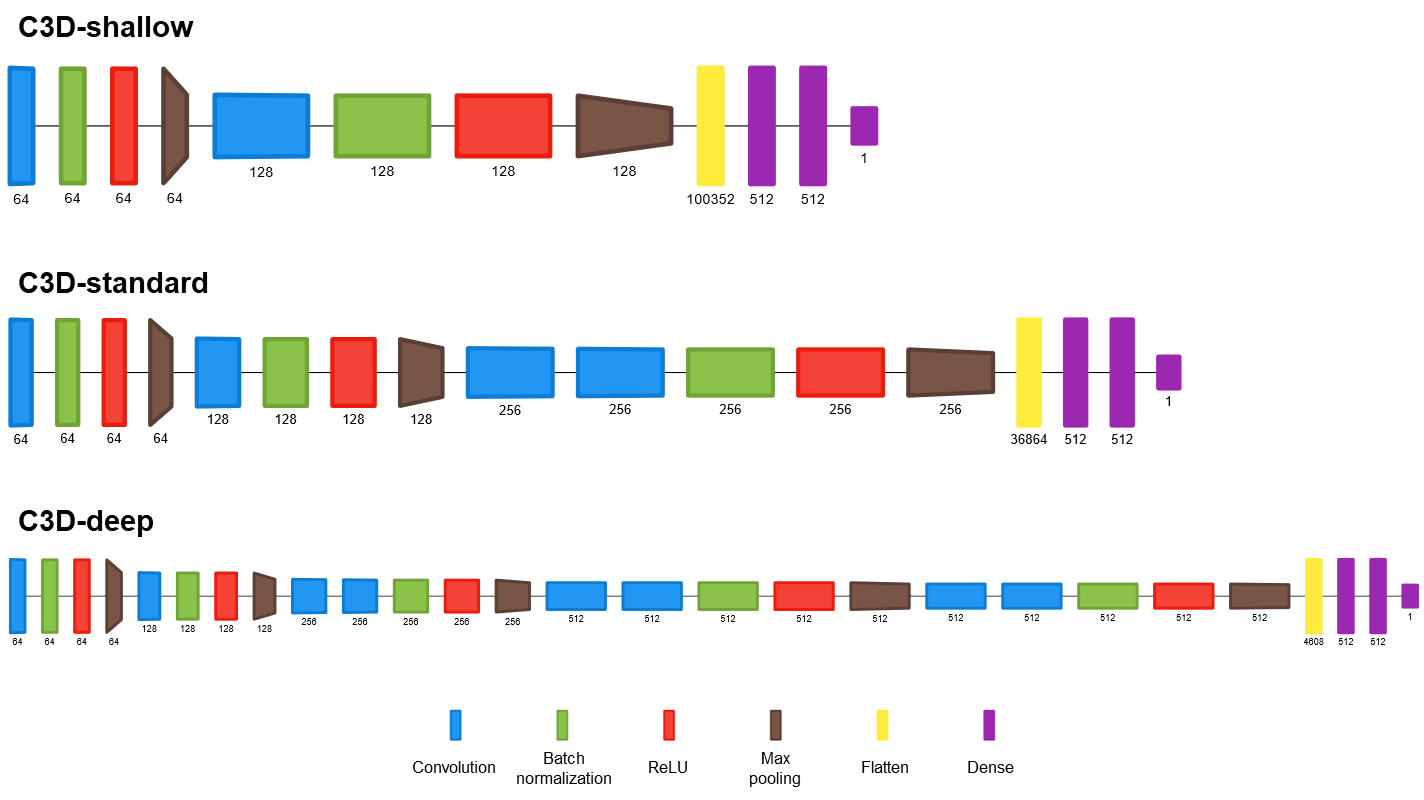


Figure S1: The CNN architectures. The shallow CNN comprised two convolutional layers, while the standard CNN and the proposed CNN classifier had four convolutional layers. The deep CNN had eight convolutional layers.


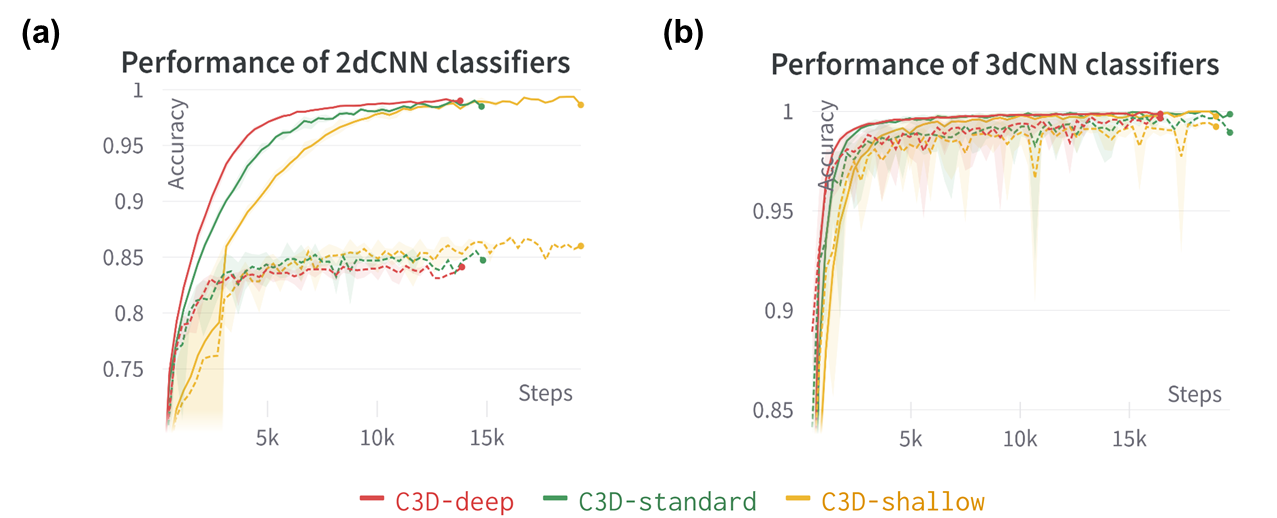


Figure S2: Training and validation learning curves of the CNN classifiers with different architectures during the training phase. The solid line represents the training accuracy, while the dotted line indicates the validation accuracy. (a) 2dCNN classifiers. (b) 3dCNN classifiers.


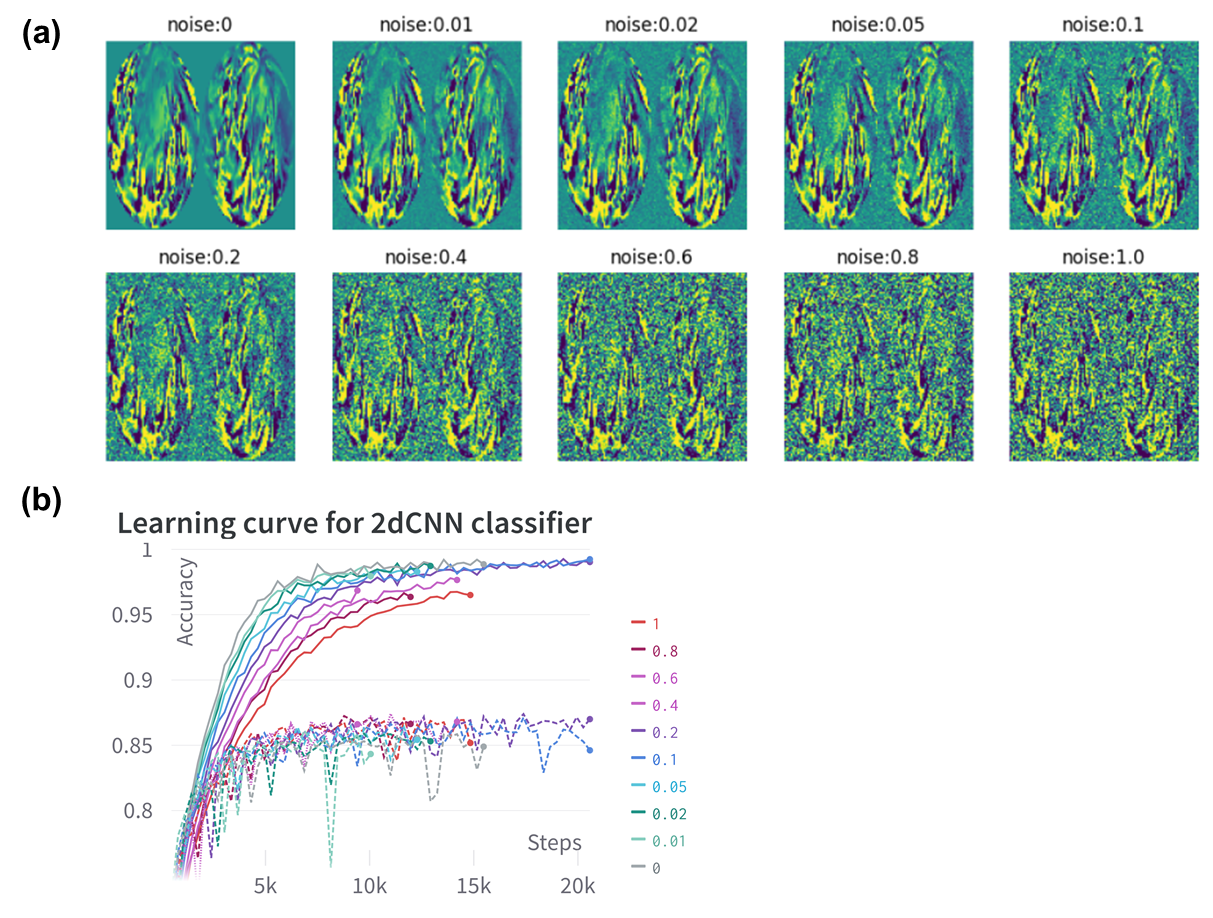


Figure S3: Classification results according to noise level. (a) Examples of input data images for various noise levels. (b) Learning curves of 2dCNN classifiers in each noise level (solid line indicates training accuracy; dotted line indicates validation accuracy).
